# Task-Based Functional Connectivity and Blood-Oxygen-Level-Dependent Activation During Within-Scanner Performance of Lumbopelvic Motor Tasks: A Functional Magnetic Resonance Imaging Study

**DOI:** 10.3389/fnhum.2022.816595

**Published:** 2022-03-02

**Authors:** Max K. Jordon, Jill Campbell Stewart, Sheri P. Silfies, Paul F. Beattie

**Affiliations:** ^1^Department of Physical Therapy, University of Tennessee at Chattanooga, Chattanooga, TN, United States; ^2^Physical Therapy Program, University of South Carolina, Columbia, SC, United States; ^3^McCausland Center for Brain Imaging, University of South Carolina, Columbia, SC, United States

**Keywords:** task-based functional connectivity, lumbopelvic, motor control, spine, movement

## Abstract

There are a limited number of neuroimaging investigations into motor control of the lumbopelvic musculature. Most investigation examining motor control of the lumbopelvic musculature utilize transcranial magnetic stimulation (TMS) and focus primarily on the motor cortex. This has resulted in a dearth of knowledge as it relates to how other regions of the brain activate during lumbopelvic movement. Additionally, task-based functional connectivity during lumbopelvic movements has not been well elucidated. Therefore, we used functional magnetic resonance imaging (fMRI) to examine brain activation and ROI-to-ROI task-based functional connectivity in 19 healthy individuals (12 female, age 29.8 ± 4.5 years) during the performance of three lumbopelvic movements: modified bilateral bridge, left unilateral bridge, and right unilateral bridge. The whole brain analysis found robust, bilateral activation within the motor regions of the brain during the bilateral bridge task, and contralateral activation of the motor regions during unilateral bridging tasks. Furthermore, the ROI-to-ROI analysis demonstrated significant connectivity of a motor network that included the supplemental motor area, bilateral precentral gyrus, and bilateral cerebellum regardless of the motor task performed. These data suggest that while whole brain activation reveals unique patterns of activation across the three tasks, functional connectivity is very similar. As motor control of the lumbopelvic area is of high interest to those studying low back pain (LBP), this study can provide a comparison for future research into potential connectivity changes that occur in individuals with LBP.

## Introduction

Neuroimaging investigations into motor control have typically focused on either upper extremity or distal lower extremity movements (Grefkes et al., 2008; Grooms et al., 2019; Vinehout et al., 2019; Criss et al., 2020). While these investigations have provided great insight into the motor control of the extremities, relatively little is known about brain activation during motor control for the lumbopelvic region. Investigations into trunk control have either relied on examining non-voluntary, postural corrections to perturbations from the extremities or have used transcranial magnetic stimulation (TMS) (Matthews et al., 2013; Jean-Charles et al., 2017). When used in conjunction with electromyography (EMG), single pulse TMS excites pyramidal neurons within the motor cortex which results in a measurable muscular contraction at the targeted site (Goss et al., 2012). While studies using TMS have provided insight into the neural correlates of lumbopelvic motor control in individuals with and without low back pain (LBP) (Tsao et al., 2008, 2011), this approach is limited for several reasons. First, the presence of pain can alter TMS findings in ways that are unpredictable (Hodges and Tucker, 2011). For example, while there is some evidence demonstrating that motor-evoked potentials (MEPs) increase during local muscle pain (Fadiga et al., 2004), several studies have shown that MEPs can either decrease (Valeriani et al., 1999; Farina et al., 2001; Martin et al., 2008), or stay the same (Romaniello et al., 2000). This variability in findings may be due to the fact that activity within a single muscle can be redistributed in order to protect the body part that is in pain (Hodges and Tucker, 2011). Therefore, findings of either increased or decreased excitability could be influenced simply by slight changes in the placement of the EMG electrode. Second, functional trunk movements require the utilization of multiple muscles working in concert with the sensory feedback. By design, TMS can only assess a single muscle at a time thus limiting its scope in investigating functional movements using multiple synergist muscle groups and sensory feedback to control volitional movements. Lastly, studies assessing motor control using TMS have only assessed the primary motor cortex and not other regions (e.g., those responsible for motor planning or proprioception) of the brain, such as premotor cortices, which might hold important insights into the motor control of the lumbopelvic region.

To better understand the neural control of the trunk, our team developed a protocol which engages the musculature of the lumbopelvic region within the confines of the MRI scanner (Silfies et al., 2020). In a previous preliminary study, modified bilateral and unilateral bridging movements activated numerus trunk muscles including the lumbar multifidus, erector spinae, external obliques, internal obliques, and rectus abdominus; hip muscles were also active (gluteus maximus, hamstrings) with greater activation on the side of movement (e.g., left gluteus and hamstrings during left bridge). During performance of the modified bridging movement, activation was recorded in a bilateral sensorimotor network that included the supplemental motor area (SMA), precentral gyrus (PreCG), postcentral gyrus (PostCG), putamen, parietal operculum, and the superior parietal lobule. During bilateral bridging, brain activation was present in both hemispheres, however, during unilateral bridging, activation was more localized to the hemisphere contralateral to movement. Overall, this previous preliminary study found that it was feasible to collect fMRI data during lumbopelvic motor tasks without excessive head movement. However, functional connectivity during the lumbopelvic tasks was not assessed in that study. A better understanding of the functional connectivity during lumbopelvic tasks could elucidate the functional integration of separate brain regions that might not be observable by looking exclusively at the change in the BOLD signal (Rao et al., 2008). Therefore, the purpose of this study was twofold. First, we aimed to validate the results of the initial feasibility study with a separate larger cohort of participants. Second, we investigated task-based functional connectivity between the sensorimotor regions during bilateral and unilateral bridging. We hypothesized that (1) the whole brain activation patterns of our study would be similar to that of the preliminary paper with bilateral activation for the bilateral bridge and contralateral activation for the unilateral bridging tasks, and (2) functional connectivity would demonstrate unique network connectivity which reflect the different sensorimotor demands of each bridging task.

## Materials and Methods

### Subjects

Twenty-one individuals were recruited to participate in this study. However, one participant was removed due to low-signal amplitude while another participant exhibited abnormal brain morphology and was unable to participate in the study. This left a total of 19 participants [12 female, age 28 ± 3.9 years, range 21–37 (Table 1)] who completed the study. After giving informed consent, participants underwent MRI safety screening to ensure they were safe to participate in the study. Inclusion criteria were: (1) being right-hand dominant; (2) being between the ages of 18–60; (3) no history of activity limiting LBP; (4) no history of inflammatory joint disease or cancer; and (5) no contraindications for undergoing MRI. Handedness was determined by the Edinburgh Handedness Inventory which also assesses for footedness (Oldfield, 1971). All of our participants were right footed. Approval for this study was given by the University of South Carolina Institutional Review Board. This data was collected as part of a larger randomized control trial which was registered with ClinicalTrials.gov (ClinicaTrials.gov ID NCT02828501) prior to the recruitment of the first participant.

**TABLE 1:** Participant demographics.

N (Female)	Average age	Age range	Weight (Lbs)	Height (in)
19 (12)	28 (3.9)	21–37	158 (40)	68 (5.2)

*Numbers in parentheses for average age, age range, weight, and height indicate standard deviation.*

### Motor Tasks Protocol

**FIGURE 1:**
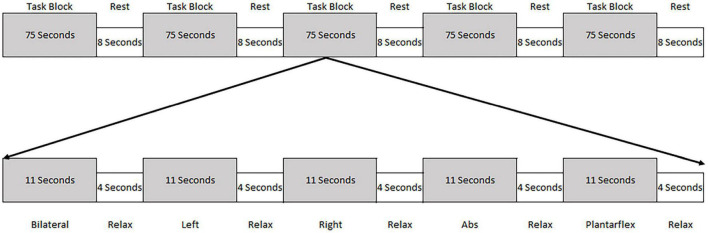
Schematic of the task block. Each task block consisted of the performance of five tasks (each for 11 s) followed by a 4 s relaxation period. The order of the tasks were randomized within the task block. Following each task block the participants were given 8 s of rest.

Participants were trained in five motor tasks prior to undergoing fMRI. The tasks included a modified bridging movement where participants pushed the back of the left knee (left bridge), right knee (right bridge), or both knees (bilateral bridge) into a firm 22 cm bolster to slightly unweight their hips without lifting them. The reason these tasks were chosen was twofold. First, our previous work demonstrated that they recruited the lumbopelvic musculature without resulting in excessive head movement (Silfies et al., 2020). Second, these tasks resemble exercises that engage muscles in areas that are commonly painful and weak in people with LBP. Two tasks, abdominal tightening and ankle plantarflexion, were also performed but were not the focus of this paper. In order to minimize the potential for physiological noise in the Blood-oxygen-level-dependent (BOLD) response, the participants were instructed to keep their head and upper body still, breathe normally, and to just slightly unweight the hips. Training for each task was done both inside and outside the MRI to familiarize the participant with the scanning environment. A block design was utilized where each motor task was performed in random order for 11 s with a 4 s relaxation period following each task. After each task block, there was an 8 s rest block where the participants were instructed to relax. This sequence was repeated six times per run, with each participant completing two runs. This led to a total of 132 s of each task being performed during the study (Figure 1).

### Functional Magnetic Resonance Imaging Acquisition Parameters

Data were collected on a 3T Siemens Prisma scanner using a 20-channel head coil (502 volumes; 58 axial slices; 2.5 mm thick; TR = 1,000 ms; TE = 37 ms; matrix 64 × 64 voxels; flip angle = 61; 220 mm × 220 mm FOV). A sagittal T1-weight MPRAGE protocol was used to acquire high-resolution structural images (192 slices; 1 mm thick; TR = 2,250 ms; TE = 4.11 ms; matrix = 1 mm × 1 mm × 1 mm; 256 × 256 FOV). The task order was recorded and the instructions were delivered to the participants using EPrime (Psychology Software Tools, Inc., Sharpsburg, PA, United States). Throughout data collection, participants were visually monitored to ensure they were performing the correct task.

### Data Pre-processing

All data were processed using SPM 12 (Wellcome Department of Cognitive Neurology, London, United Kingdom) implemented in MATLAB R2017a (Mathworks, Natick, MA, United States). Initially, for each run, every volume was realigned to the first and unwarped. Using the anatomical scan, the mean image for each participant was then normalized to standard Montreal Neurological Institute (MNI) space. Once the normalization was completed, the parameters were applied to each volume in the functional run and data were resampled to 2 mm × 2 mm × 2 mm voxels. Smoothing was then applied using an isotropic Gaussian kernel 8 mm × 8 mm × 8 mm full width at half maximum. Head motion was then assessed for all analyzed data using the Artifact Detection Tool toolbox.^1^ The first derivative of the head motion was used to screen for excessive head motion, and all outliers (defined as a greater than 2 mm difference from the previous volume) were de-weighted during the statistical analysis (mean number of outliers per run = 2, ranged from 0 to 8).

### Statistical Analysis

#### Functional Magnetic Resonance Imaging Whole Brain Analysis

First-level analysis was performed using a general linear model for each participant (Friston et al., 1995; Worsley and Friston, 1995). Contrast maps were calculated for each task period vs. rest using the first derivative of head motion for all six directions as a regressor of no interest. The contrast maps for each of the bridging tasks were then moved to a second-level random effects analysis. A group analysis using a factorial design was performed with a factor for condition (left, right, and bilateral bridge). We analyzed the main effect for each condition, the comparison of one condition against another, as well as the combined effect for all bridging tasks. Group-level results were thresholded at a *p*-value less than 0.05 that was corrected for multiple comparisons using familywise error (FWE).

#### Functional Connectivity Analysis

We originally planned to select regions of interest (ROI) based on the results of our previous work using the same motor tasks (Silfies et al., 2020). However, the whole brain analysis found no activation peaks within the PostCG and consistent peaks within the cerebellum. Therefore, we choose the following ROIs to represent a sensorimotor network likely to be utilized during the bridging tasks based on the results of our whole brain analysis: bilateral Precentral Gyrus (PreCG), bilateral Cerebellum, and supplementary motor area (SMA). Using MarsBAR, we created a 5 mm radius sphere centered on the maximum peak of activation found in the group mean bridge analysis. This resulted in ROIs centered on the following MNI coordinates: Left PreCG (−14, −28, 68), Right PreCG (14, −28, 66), Left Cerebellum (−8, −42, −14), Right Cerebellum (6, −42, −16), and SMA (0, −16, 64).

Functional connectivity during movement was analyzed using the CONN toolbox (Whitfield-Gabrieli and Nieto-Castanon, 2012). Each participant’s data was imported into the toolbox along with the task onsets and durations. Confounds were then removed *via* CONN’s CompCor algorithm for physiological noise to reduce their effect on the functional connectivity values. A GLM approach was used for the ROI-to-ROI connectivity analysis. A bivariate correlation was computed separately on the individual’s BOLD time series between each pair of ROIs; correlation coefficients were then transformed to Fisher’s *Z* scores to meet the assumptions of normality (Whitfield-Gabrieli and Nieto-Castanon, 2012). The Fisher-Z transformed correlations were then extracted from the first-level analysis using MatLab and imported into SPSS (IBM SPSS Statistics for Windows, Version 25.0). A one-Sample’s *t*-test was performed to determine if the correlations between each ROI pair were significantly different from 0 using the Holm’s sequential Bonferroni procedure to correct for multiple comparisons (Eichstaedt et al., 2013). Then, an ANOVA with repeated measures (rmANOVA) was used to determine if the correlations between the different ROIs differed based on the task performed. For the rmANOVA, significance was determined using an α = 0.05 with a Bonferroni correction.

## Results

### Activation During Lumbopelvic Motor Task Performance

**FIGURE 2:**
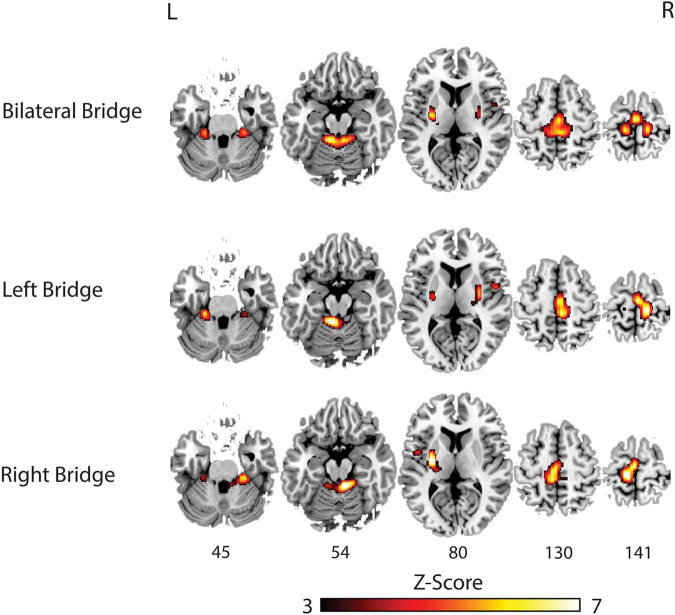
Group analysis of brain activation for each task compared to rest. L, **Left**; R, **Right**.

Brain activation during each motor task is shown in Figure 2 and Table 2. Activation during the bridging tasks included multiple areas in the sensorimotor network consistent with Silfies et al. (2020) and included the PreCG, SMA, Cerebellum, and Putamen. Motor cortex activation was primarily located in the medial regions of the sensorimotor cortex (Figure 2) consistent with the somatotopic organization of this region (Asavasopon et al., 2014; Saby et al., 2015). As expected, activation was present in both hemispheres during bilateral bridging task while activation was predominantly located in the contralateral cerebral hemisphere (i.e., right motor regions during left bridge and left motor regions during right bridge) and the ipsilateral cerebellum during unilateral bridging tasks.

**TABLE 2:** Whole brain BOLD response of relative activation compared to rest.

Comparison	Cluster	No. of voxels	P FWE-corr	Peak-*Z*	MNI location, mm			Structural regions
					*X*	*Y*	*Z*	
Bilateral bridge > Rest	1	200	<0.001	6.04	−10	−40	−16	L Cerebellum
				5.34	6	−42	−16	Vermis
				4.92	16	−38	−20	R Cerebellum
	2	497	<0.001	5.89	0	−16	66	Supplemental motor area
				5.33	14	−28	66	R Precentral Gyrus
				5.28	−12	−30	68	L Precentral Gyrus
	3	37	0.007	5.55	−28	−10	10	L Putamen
Left bridge > Rest	1	230	<0.001	6.6	−8	−42	−16	L Cerebellum
				5.19	−22	−32	−28	L Cerebellum
	2	616	<0.001	6.20	12	−28	70	R Thalamus
				3.05	2	−16	64	R Supplemental motor area
				5.58	6	−32	58	R Precentral Gyrus
	3	25	0.012	4.95	30	−10	6	R Putamen
Right bridge > Rest	1	146	<0.001	6.79	−28	−10	10	L Putamen
				4.75	−24	−22	14	L Thalamus
	2	554	<0.001	6.42	−12	−28	70	L Precentral Gyrus
				5.97	−4	−20	64	L Supplemental motor area
				5.51	−6	−34	58	L Precuneus
	3	238	0.016	6.41	8	−42	−18	R Cerebellum
				5.20	24	−32	−28	R Cerebellum

*Comparisons of each task against rest. All clusters were significant at p < 0.05 with familywise error correction (FWE-corr) for analysis. In both unilateral bridging tasks, the location of the peak voxel within the somatosensory regions were located in the contralateral hemisphere.*

*No. of voxels, number of 2 mm^3^ voxels in the cluster; Peak-Z, peak Z-value within the cluster; L, Left; R, Right; Rest, rest condition no movement.*

**FIGURE 3:**
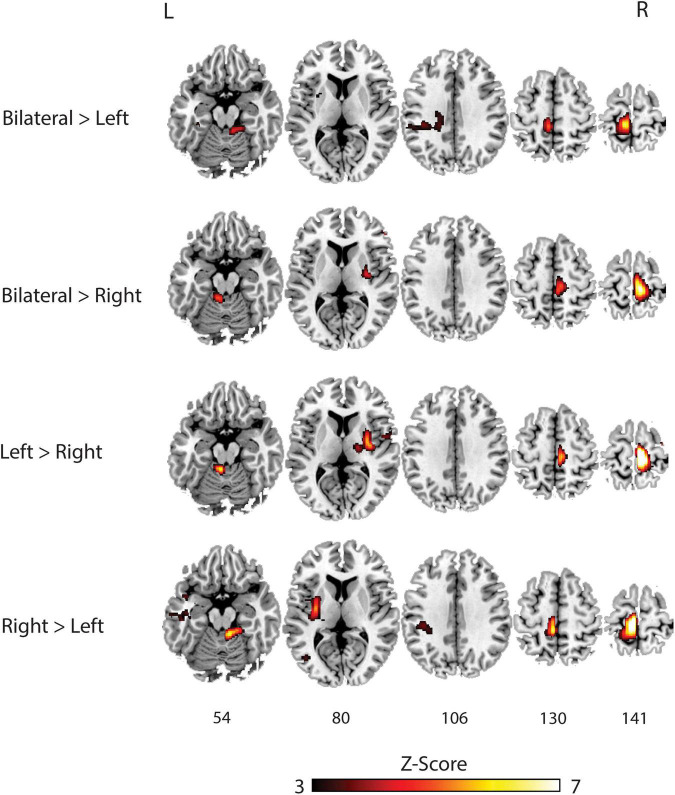
Group analysis of brain activation for each task compared against each other. L, **Left**; R, **Right**.

Table 3 and Figure 3 outlines the differences in activation between the tasks. When compared to the left bridging task, the bilateral bridge had greater activation in the left PreCG. Similarly, when compared to the right bridge, the bilateral bridge had greater activation in the right PreCG. When comparing the unilateral bridging tasks against one another, both tasks demonstrated greater activity in the contralateral PreCG and Putamen, as well as greater activity in the ipsilateral Cerebellum. However, when comparing the right bridge to the left bridge, there was significantly greater activation in the left PostCG and Insula.

**TABLE 3:** Whole brain BOLD response of comparative bridging tasks.

Comparison	Cluster	No. of voxels	P FWE-corr	Peak-Z	MNI location, mm			Structural regions
					*X*	*Y*	*Z*	
Bilateral > Left	1	196	<0.001	5.63	−8	−32	70	L Precentral Gyrus
Bilateral > Right	1	474	<0.001	6.41	10	−28	74	R Precentral Gyrus
Left > Right	1	694	<0.001	7.65	10	−28	74	R Precentral Gyrus
	2	93	0.001	8.76	−10	−38	−22	L Cerebellum
	3	51	0.005	5.08	32	−10	6	R Putamen
Right > Left	1	583	<0.001	7.21	−8	−30	70	L Precentral Gyrus
				6.24	−6	−36	62	L Postcentral Gyrus
	2	230	<0.001	5.82	−30	−22	18	L Insular Cortex
				5.48	−28	−8	12	L Putamen
	3	75	0.002	5.69	10	−40	−20	R Cerebellum

*Results from comparing each bridging task against one another. All clusters were significant at p < 0.05 with familywise error correction (FWE-corr) for analysis.*

*No. of voxels, number of 2 mm^3^ voxels in the cluster; Peak-Z, peak Z-value within the cluster; L, Left; R, Right.*

### Connectivity During Task Performance

**FIGURE 4:**
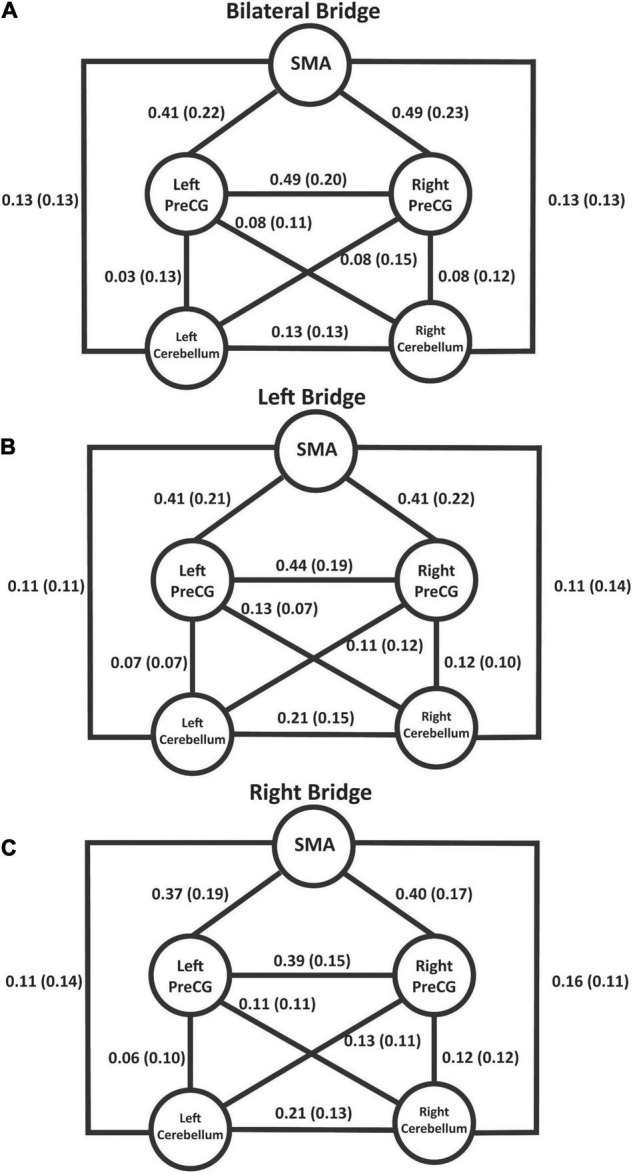
Schematic of the proposed sensorimotor network during the bridging tasks. Values represent Fisher’s-Z transformed correlation coefficients with standard deviation in parentheses. With the exception of left and right PreCG to left cerebellum during the bilateral bridge task, all values were significant at *p* < 0.05 after correction using the Holm’s sequential Bonferroni procedure (Eichstaedt et al., 2013). **(A)** Connectivity values during the bilateral bridge task. **(B)** Connectivity values during the left bridge task. **(C)** Connectivity values during the right bridge task. SMA, supplemental motor area; PreCG, precentral gyrus.

Figure 4 summarizes the connectivity values within the proposed sensorimotor network during the bridging tasks. The individual *t*-tests demonstrated that the only correlations that were not significant at the *p* = 0.05 level after correction were the connections between the left PreCG and the left cerebellum (*p* = 0.415) and the right PreCG and the left cerebellum (*p* = 0.052) during the bilateral bridging tasks. All other connections were significant. The results of the rmANOVAs revealed some significant differences in the connectivity between the tasks. First, the connectivity between the right PreCG and left PreCG during the bilateral bridging task was significantly higher when compared to the right bridging task (*z* = 0.491 vs. *z* = 0.395; *p* = 0.032). This difference was not observed when comparing the bilateral to the left bridging task. Additionally, the connectivity between the right PreCG and the SMA during the bilateral bridge was significantly higher when compared to the right bridging task (*z* = 0.493 vs. *z* = 0.396; *p* = 0.009).

## Discussion

To our knowledge, this is the first study to exam functional connectivity during volitional movements of the lumbopelvic region. The primary aims of this study were to validate the results of a previous investigation (Silfies et al., 2020) and to examine functional connectivity in the sensorimotor network during lumbopelvic motor tasks. Similar to the previous study, robust activation in the medial sensorimotor regions were observed during motor tasks which involved the lumbopelvic musculature. Additionally, during the unilateral bridging tasks activation was shifted toward the contralateral hemisphere, whereas during the bilateral bridging task activation was present in both hemispheres. The functional connectivity analysis demonstrated significant connectivity between each of the ROIs for each of the bridging tasks, with some differences in connectivity exhibited between the right and bilateral bridging tasks.

### Sensorimotor Activation During Lumbopelvic Motor Tasks

As hypothesized, during lumbopelvic bridging tasks we found strong activation in the medial motor areas of the brain, consistent with a previous preliminary study (Rao et al., 2008). While the current study utilized tasks which focused on engagement of the lumbopelvic musculature, previous literature investigating cortical activation during other lower limb tasks supports the general activation patterns we found (Mehta et al., 2012). Studies that included unilateral ankle (Debaere et al., 2001; Kapreli et al., 2006, 2007; Cunningham et al., 2013), knee (Fink et al., 1997; Luft et al., 2002; Kapreli et al., 2006, 2007), and toe (Kapreli et al., 2006, 2007) movements have consistently reported activation in the SMA, PreCG, and Cerebellum. Furthermore, previous studies have found that the PreCG is somatotopically organized with the feet represented relatively medially and the hands represented relatively laterally (Rao et al., 1995; Kapreli et al., 2007; Plow et al., 2010; Cunningham et al., 2013; Weiss et al., 2013). Overall, the bridging tasks used in the current study activated a medial sensorimotor network, suggesting this protocol provides an approach to examine the neural correlates of lumbopelvic motor control.

One observation that was different for this study compared to our previous one was the amount of lateralization that occurred with the unilateral bridging tasks. Consistent with our previous study, the bilateral bridge resulted in bilateral activation; however, the unilateral bridging tasks in this study resulted in more lateralized activation. Specifically, the unilateral bridging tasks resulted in activation of the contralateral PreCG, SMA, Thalamus, and Putamen, with ipsilateral cerebellar activation. This pattern resembles previous work investigating sensorimotor activation during movement (Grefkes et al., 2008). However, our previous study reported activation occurring across both brain hemispheres during unilateral bridging. One reason for the difference may be due to the slight differences in the task hold time: in our previous study participants held the tasks for 14 s, whereas in our current study they only held the tasks for 11 s. While the difference is small, the extra 3 s might have been enough to necessitate additional recruitment of the trunk musculature in order to prevent fatigue, thus obscuring the distinct hemispheric pattern we observed in the present study. An alternative explanation could be in the total time engaged in each task. In the previous study, the participants performed 84 s of each task, whereas in our current study this was increased to 132 s. This more than 50% increase in task time might have resulted in the more specific activation patterns that were observed. Regardless, this study demonstrated that the lumbopelvic protocol used in the current study is able to delineate different patterns of activation based on the unique demands of the three bridging tasks.

### Functional Connectivity During Lumbopelvic Motor Tasks

With limited exceptions, the sensorimotor network we described was significantly connected during the performance of lumbopelvic tasks. Similar to previous work in the upper and lower limb, the bilateral bridging task resulted in interhemispheric connectivity (Grefkes et al., 2008; Vinehout et al., 2019). However, our findings did not fully support our hypothesis. We hypothesized that the unilateral bridging tasks would demonstrate unique connectivity patterns which reflected the specificity of the task. While this was evident in whole brain activation, we found that the pattern of connectivity did not differ between the unilateral bridging tasks, and only minimally so between the bilateral and right bridging tasks. This is inconsistent with previous work investigating differences in unilateral vs. bilateral tasks (Grefkes et al., 2008; Vinehout et al., 2019). For example, Vinehout et al. (2019) examined differences in lower limb task-based functional connectivity in asymptomatic individuals and individuals who had a stroke. They reported that the strength of the functional connections between each of the ROIs was modulated by the tasks. One possible explanation of why our findings were inconsistent with this previous work using extremity movement could be due to the tasks that were used in each study. In the study by Vinehout et al., the bilateral movement was a multi-joint pedaling task that required the coordination of multiple segments; whereas the unilateral task was tapping of the foot, which would require the use of only a single joint. While our tasks incorporated both unilateral and bilateral lumbopelvic movements, all the tasks required the coordination of multiple segments. Therefore, the uniformity of the connectivity values in our study might reflect the complexity of the movement and the higher demands for sensorimotor integration of multi-segmental motor tasks (Vinehout et al., 2019).

This hypothesis is further supported by work that has been done in the upper extremity as well. Prior evidence has shown that unilateral hand opening/closing tasks results in connectivity within the contralateral hemisphere, while bilateral hand opening/closing tasks increases the interhemispheric connectivity (Grefkes et al., 2008). However, Wilkins et al. found that when performing a unilateral hand grasping task, by increasing the complexity of the activity and having the participants coordinate motion between multiple joints of the same limb there was an increase in interhemispheric communication (Wilkins and Yao, 2020). This increase in the interhemispheric communication was absent with a simple hand opening task. Thus, the integration of movement from multiple joints might also explain why there was little difference in interhemispheric connectivity between our bilateral and unilateral lumbopelvic tasks. By utilizing lumbopelvic as opposed to upper or lower limb tasks, our results support the notion that an increase in interhemispheric connectivity is related to the complexity of movement independent of the bilateral or unilateral nature of the task being performed.

One contributing factor to the complexity of the lumbopelvic task could be the bilateral recruitment of the trunk musculature required to stabilize the spine during the modified bridging task (Yoon et al., 2018), whereas no such stabilization is required during foot tapping or performance of simple upper extremity motor tasks. Performing a modified bridge is a complex motor task that requires coordination across the lumbopelvic musculature in order to stiffen the spine and maintain balance while the pelvis is being lifted from the mat (Kim et al., 2013; Czaprowski et al., 2014; Yoon et al., 2018). While the activation patterns in the whole brain analysis were different depending on the task performed, it may be that functional connectivity reflects the coordination and communication required between the bilateral trunk musculature which would be needed regardless of the task (Rao et al., 2008). Thus, while the participants exhibited unique activation patterns during the whole brain analysis, the differences in the functional connectivity could be minimal. Strong structural connections between these regions could also drive the similarity in functional connectivity across the tasks (Ansari et al., 2011). The SMA and PreCG work together to help facilitate movement. The SMA, which is largely devoted to movement planning and early motor preparation has structural connections with the PreCG (Ruddy et al., 2017). Considering the strong structural connections and synergies in function, our results fit well within the established literature.

### Implications for Low Back Pain Research

There have been numerous investigations into both the functional connectivity and brain activation during motor tasks involving the hand and upper extremities (Grefkes et al., 2008; Xu et al., 2014; Coombes and Misra, 2016). However, more investigation is needed into the lumbopelvic musculature as this region is implicated in those with chronic low back pain (cLBP). Previous research has demonstrated that cLBP results in specific cortical changes linked to the lumbopelvic region; during both muscle (Tsao et al., 2008, 2011; Schabrun et al., 2015) and cutaneous stimulation (Flor et al., 1997; Hotz-Boendermaker et al., 2016). Furthermore, biomechanical research has suggested deficits in the lumbopelvic motor control in individuals with cLBP (Hodges and Richardson, 1996; Henry et al., 2006; Silfies et al., 2009; Jones et al., 2012; Sung et al., 2015). Therefore, a better understanding of the processes behind the motor control of the lumbopelvic musculature could potentially lead to better therapies in the treatment of those with LBP. By providing functional connectivity data in individuals devoid of pain, the results of this investigation can provide a comparison for future research into potential connectivity changes in individuals with cLBP. Furthermore, this protocol provides researchers another method by which to examine motor control and the effects of different interventions in individuals with cLBP.

### Limitations

Unlike previous research using lower extremity tasks, we did not incorporate external stabilization devices to reduce motion artifact and control movement (Debaere et al., 2001; Kapreli et al., 2006, 2007; Newton et al., 2008). While stabilizing the joint may decrease task-related head movement, this isolation may influence the findings. There is an inherent motor variability during movement performance (Balasubramaniam et al., 2000) and the ability to compensate for this variation is vital for optimal feedback control (Todorov and Jordan, 2002). Supplementing joint support during a task may reduce the ability to detect changes in individuals with chronic pain who demonstrate movement impairment (Hodges and Richardson, 1996; Henry et al., 2006; Silfies et al., 2009; Jones et al., 2012; Sung et al., 2015). Stabilizing joint motion appears to improve sensorimotor function as well (Wu et al., 2001; de Vries et al., 2017; Smalley et al., 2018), and may inadvertently diminish differences that may be found between asymptomatic individuals and individuals with musculoskeletal disorders such as cLBP (Tsao et al., 2008, 2011; Schabrun et al., 2015). As such, lumbopelvic motor tasks that are unencumbered by external support may be an important approach for elucidating the cortical changes associated with cLBP. Furthermore, with an average of 2 out of 765 volumes being removed for excessive motion, our task did not seem to create excessive artifact. This in in line with our previous work which found that this specific motor protocol resulted in minimal head movement (Silfies et al., 2020).

Another potential limitation of our study is that the method of analyzing connectivity we chose does not allow for insights into directionality of the network, which could help clarify the modulation of activity between our different ROIs. Future studies could consider the interaction between these regions using an effective connectivity analysis approach.

## Conclusion

We examined brain activation and functional connectivity during the performance of unsupported bilateral and unilateral lumbopelvic motor tasks. Robust activation patterns were observed in the motor network and differences were observed depending on the task being performed. Within our constrained motor network of the PreCG, Cerebellum, and SMA we found extensive connectivity between these regions across tasks. This study helps build a foundation for future investigations designed to examine the changes in the neural correlates of movement in individuals with LBP and inform the development of intervention approaches.

## Data Availability Statement

The datasets presented in this study can be found in online repositories. The names of the repository/repositories and accession number(s) can be found below: All relevant data are available on Mendeley with the DOI: 10.17632/v9yhyrhhp5.1.

## Ethics Statement

The studies involving human participants were reviewed and approved by the University of South Carolina Institutional Review Board. The patients/participants provided their written informed consent to participate in this study.

## Author Contributions

MJ: conceptualization, methodology, formal analysis, investigation, writing—original draft, writing—review and editing, and funding acquisition. JS: formal analysis, writing—original draft, and writing—review and editing. SS: conceptualization, methodology, investigation, and writing—review and editing. PB: conceptualization, writing—review and editing, and funding acquisition. All authors contributed to the article and approved the submitted version.

## Conflict of Interest

The authors declare that the research was conducted in the absence of any commercial or financial relationships that could be construed as a potential conflict of interest.

## Publisher’s Note

All claims expressed in this article are solely those of the authors and do not necessarily represent those of their affiliated organizations, or those of the publisher, the editors and the reviewers. Any product that may be evaluated in this article, or claim that may be made by its manufacturer, is not guaranteed or endorsed by the publisher.

## Footnotes

1
https://www.nitrc.org/projects/artifact_detect/
